# Increased expression of kisspeptin and GnRH forms in the brain of scombroid fish during final ovarian maturation and ovulation

**DOI:** 10.1186/1477-7827-10-64

**Published:** 2012-08-27

**Authors:** Sethu Selvaraj, Hajime Kitano, Masafumi Amano, Hirofumi Ohga, Michio Yoneda, Akihiko Yamaguchi, Akio Shimizu, Michiya Matsuyama

**Affiliations:** 1 Laboratory of Marine Biology, Kyushu University, Fukuoka, 812-8581, Japan; 2 School of Marine Biosciences, Kitasato University, Sagamihara, Kanagawa, 252-0373, Japan; 3 Fisheries Research Agency, Kanazawa, Yokohama, 236-8648, Japan

**Keywords:** Kisspeptins, Kiss, GnRH, Mackerel, Brain, Pituitary, Spawning cycle

## Abstract

**Background:**

Kisspeptins (Kiss) are prime players in the control of reproductive function through their regulation of gonadotropin-releasing hormone (GnRH) expression in the brain. The experimental scombroid fish, chub mackerel (*Scomber japonicus*) expresses two *kiss* (*kiss1* and *kiss2*) and three *gnrh* (*gnrh1*, *gnrh2*, and *gnrh3*) forms in the brain. In the present study, we analyzed expression changes of *kiss* and *gnrh* mRNAs in the brain and corresponding GnRH peptides in the brain and pituitary during final ovarian maturation (FOM) and ovulation.

**Methods:**

Female fish possessing late vitellogenic oocytes were injected with GnRH analogue to induce FOM and ovulation. Fish were observed for daily spawning activities and sampled one week post-injection at germinal vesicle migration (GVM), oocyte hydration, ovulation, and post-ovulatory time periods. Changes in relative mRNA levels of *kiss* and *gnrh* forms in the brain were determined using quantitative real-time PCR. Changes in GnRH peptides in the brain and pituitary were analyzed using time-resolved fluoroimmunoassay.

**Results:**

Both *kiss1* and *kiss2* mRNA levels in the brain were low at late vitellogenic stage and increased significantly during the GVM period. However, *kiss1* mRNA levels decreased during oocyte hydration before increasing again at ovulatory and post-ovulatory periods. In contrast, *kiss2* mRNA levels decreased at ovulatory and post-ovulatory periods. Levels of *gnrh1* mRNA in the brain increased only during post-ovulatory period. However, levels of *gnrh2* and *gnrh3* mRNAs were elevated during GVM and then, decreased during oocyte hydration before increasing again at ovulatory period. During post-ovulatory period, both *gnrh2* and *gnrh3* mRNA levels declined. Peptide levels of all three GnRH forms in the brain were elevated during GVM and oocyte hydration; their levels were significantly lower during late vitellogenic, ovulatory, and post-ovulatory periods. In contrast, pituitary GnRH peptide levels did not show any significant fluctuations, with the GnRH1 peptide levels being many-fold higher than the GnRH2 and GnRH3 forms.

**Conclusion:**

The results indicate increased expression of multiple Kiss and GnRH forms in the brain and suggest their possible involvement in the regulation of FOM and ovulation in captive female chub mackerel.

## Background

In vertebrates, including teleosts, reproductive processes are regulated by the precise coordination of neuroendocrine hormones acting through the brain-pituitary-gonad (BPG) axis. A neurohormone, gonadotropin-releasing hormone (GnRH), plays a central role by stimulating the synthesis and release of the pituitary gonadotropins (GtHs). These pituitary GtHs, follicle-stimulating hormone (FSH) and luteinizing hormone (LH), act on the gonads to stimulate steroidogenesis, which is responsible for progression of ovarian growth and maturation [1,2]. However, in recent years, kisspeptins, a member of the RF-amide family, have been shown to act as an upstream endogenous regulator of GnRH neurons in mammals [3,4]. Recent studies indicate that their role in teleostean fish is also conserved [5,6]. Kisspeptins primarily act at the level of GnRH neurons, which express kisspeptin receptor (GPR54 or Kiss1r) [7,8].

Studies in teleosts have revealed the presence of multiple kisspeptin forms (Kiss1, Kiss2) in the brain [6]. Moreover, teleosts brain expresses multiple GnRH forms (GnRH1, GnRH2, and GnRH3) with one or two forms regulating pituitary function [9,10]. These multiplicities have complicated our understanding of their physiological roles in the gonadal growth and maturation in teleosts, especially in females as they exhibit different forms of reproductive dysfunctions when reared in captivity [11].

The experimental scombroid fish model, chub mackerel (*Scomber japonicus*), is a multiple batch-spawning pelagic fish. It is one of the most commercially important marine fish species in Japan. This species has been targeted for aquaculture in recent years owing to a sharp decline in the wild population, high consumer demand, use in the tuna fishing industry as bait, and high early growth potential [12]. In southwestern Japan, wild-caught fish are being used for aquaculture production in sea pens [13]. However, vitellogenic females fail to undergo final ovarian maturation (FOM) and ovulation in aquaculture conditions [13,14]. Therefore, characterization and understanding of neuroendocrine pathways acting via BPG axis is critical to clarify the reproductive dysfunction in female chub mackerel [15]. Our group already standardized a protocol based on sustained GnRH analogue delivery system to induce FOM and ovulation in outdoor tanks during natural spawning season (April-June) [16]. This system allows us to sample fish at different stages of FOM and ovulation.

The chub mackerel brain expresses *kiss1* and *kiss2*. During the seasonal ovarian cycle, *kiss2* mRNA levels decrease during vitellogenic and ovarian regression stages [17]. Also, the presence of three GnRH forms, namely GnRH1, GnRH2, and GnRH3 (previously seabream GnRH, chicken GnRH-II, and salmon GnRH forms, respectively [18]) in the brain were demonstrated previously [19,20]. An increase in the pituitary peptide levels of GnRH1 was observed during ovarian growth and regression stages [20], in agreement with our immunocytochemical observation of dense GnRH1-immunoreactive (ir) fibers localized close to FSH- and LH-producing cells in the pituitary [19]. In female gilthead seabream (*Sparus aurata*), which also express three GnRH forms as that of chub mackerel, an increase in the levels of all three forms of GnRH-encoding mRNAs in the brain was reported during FOM [21]. In the present study, to clarify the possible involvement of kisspeptin and GnRH system in the regulation of FOM and ovulation, we analyzed the expression profiles of *kiss* and *gnrh* mRNAs in the brain as well as corresponding GnRH peptides in the pituitary of chub mackerel after initial administration of GnRH analogue.

## Methods

### Fish and tissue sampling

Adult chub mackerel (2-year-old) were caught from the wild using purse seine during autumn 2008 and reared for six months in sea pens at a fish farm in the Oita prefecture, Kyushu Island. During the following spawning season (April-June), fish were transferred to Fishery Research Laboratory of Kyushu University and moved into 3-ton outdoor concrete tanks circulated with running seawater. The fish were acclimated and reared under natural photoperiod and temperature. Our previous studies indicated that female chub mackerel fail to undergo FOM and ovulation spontaneously in this captive system [13,14]. An induced spawning protocol based on sustained release GnRH delivery system was adopted from previous study [16].

After 3 days of acclimation, fish were anaesthetized with 2-phenoxyethanol (100 mg/l) and females with late vitellogenic oocytes (600–650 μm in diameter) were selected by ovarian biopsy using a plastic catheter tube (2 mm internal diameter), as described previously [13,16]. Males oozing milt under gentle abdominal pressure were selected. After selection of required number of females and males, intramuscular injection with the GnRH agonist (D-Ala^6^, des-Gly^10^)-LHRH ethylamide (Sigma-Aldrich, St. Louis, USA) at 400 μg/kg body weight were performed on April 30^th^, May 13^th^, May 21^st^, and May 22^nd^, 2009 to obtain different ovarian stages, namely germinal vesicle migration (GVM), oocyte hydration (HY), ovulation (OV), and post-ovulation (POV), respectively. The sampling times were 13.00, 16.00, 20.00, and 6.00 h of the day, respectively. In all cases, injections were performed at 11.00 h. Sampling times were fixed based on our previous data on time course of FOM and ovulation in chub mackerel induced by GnRHa [16]. Fish sampling for the analysis was performed on day 8, based on previous reports showing the decline in the plasma concentration of GnRH agonist on day 5 after intramuscular injection with the GnRH agonist suspended in coconut oil in Plaice, *Pleuronectes platessa*[22,23]. The first spawning was observed 34–36 h post-injection, and subsequent daily spawning occurred between 22.00 and 24.00 h. In the following experimental system, daily spawning of chub mackerel is observed for 20–30 days during the spawning season, when the water temperature ranged between 18-23°C (Yoneda et al., unpublished observations). The late vitellogenic (LV) stage fish were sampled before the start of induced spawning experiment.

Fish used in the experiment were sacrificed in accordance with the guidelines for animal experiments proposed by the Faculty of Agriculture and Graduate Course at Kyushu University and according to the laws (No. 105) and notifications (No. 6) of the Japanese government. The fork length, body, and gonad weights of each individual were measured before tissue sampling. The brain and pituitary of each fish were removed following decapitation, snap-frozen in liquid nitrogen, and stored at −80°C until further analysis. For ovarian histological evaluation, ovary midsections from individual fish were fixed in Bouin’s solution. To analyze the changes in *kiss* and *gnrh* mRNA levels in the whole brain and GnRH peptides in the whole brain and pituitary, two experimental sets of fish samples were used (Table 1). The brain tissue from the first set was used for mRNA analysis, and the second set was used for GnRH peptide analysis. Male fish were excluded from the analysis.

**Table 1:** Fork length, body weight, and gonadosomatic index of female chub mackerel analyzed in the study period

**Analyses**	**Parameters**	**Ovarian stages**				
		**LV**	**GVM**	**HY**	**OV**	**POV**
Kiss/GnRH mRNAs	Fork length (cm)	33.6 ± 0.4	34.7±0.7	34.6±0.5	33.0±0.4	34.7±0.6
	Body weight (g)	523.6 ±24.1	637.9±54.7	692.3±38.1	522.1±13.7	591.5±26.0
	GSI (%)	7.3 ± 1.4	7.7±1.6	13.7±2.8	7.0±0.8	6.2±1.1
	n	6	6	5	6	6
GnRH peptides	Fork length (cm)	33.5 ± 0.5	34.6 ± 0.8	34.9 ± 0.5	33.2 ± 0.4	35.3 ± 1.3
	Body weight (g)	521.9 ±24.1	578.6 ± 30.0	659.9 ± 44.0	513.5 ± 19.3	694.7 ± 94.4
	GSI (%)	6.7 ± 0.8	4.5 ± 0.66	8.2 ± 1.8	6.9 ± 1.2	8.0 ± 2.7
	n	6	5	4	6	4

Values are expressed as the mean ± SEM. LV, late vitellogenesis; GVM, germinal vesicle migration; HY, hydration; OV, ovulation; POV, post-ovulation; GSI, gonadosomatic index (GSI=gonad weight/body weight without gonads x 100).

### Ovarian histology

After fixation, ovary samples from each fish were dehydrated in a series of ethanol solutions up to 100%, embedded in paraffin, and sectioned at 5–7 μm using a Leica RM 2155 rotary microtome (Leica, Germany). Sections were stained with hematoxylin and counterstained with eosin. The stained tissues were subsequently observed under a light microscope. Chub mackerel show asynchronous ovarian development, and ovarian stages were thus classified based on the developmental stages of first clutch oocytes as (1) LV, (2) GVM, (3) HY, (4) OV, and (5) POV.

### Quantitative real-time PCR analysis of *kiss* and *gnrh* mRNAs in the brain

Quantitative real-time PCR (qRT-PCR) analysis was performed on an Mx 3000P quantitative PCR system (Stratagene). Total brain RNA was extracted using ISOGEN (Nippon Gene, Japan), according to the manufacturer’s protocol. One microgram of total RNA from each brain sample was digested with DNase I (Invitrogen) and used as template for reverse transcription (RT) reaction. The cDNA synthesis was performed using Superscript III Reverse Transcriptase (Invitrogen) in a 20 μl reaction mixture containing 2.5 mM dNTP mix, random primers (100 ng/μl; Takara Bio Inc., Japan), 5X First-Strand buffer, 0.1 M DTT, and RNase H (2 units). Based on our previous report on full-length cDNA sequences [17,20], gene specific primers for chub mackerel *kiss1kiss2* (GenBank accession number: GU731672 and GU731673), *gnrh1gnrh2*, and *gnrh3* (GenBank accession number: HQ108193, HQ108194, and HQ108195) were designed from the open reading frame region of each gene using GENETYX software (Table 2) and validated with RT-PCR and agarose gel electrophoresis. Amplification of the beta (*β*)-*actin* (GenBank accession number: GU731674) was used as the endogenous reference gene to correct for differences in reverse transcription efficiency and template quantity. The qRT-PCR was performed using the Brilliant II Fast SYBR Green QPCR Master Mix (Stratagene), following the manufacturer’s protocol. The thermocycling conditions were set as 95°C for 5 min and 40 cycles of 95°C for 10 sec and 60°C for 30 sec. Dissociation curve analysis was also included; one cycle of 95°C for 1 min, 55°C for 30 sec, and 95°C for 30 sec. All transcripts were quantified using a standard curve method [24] and a previously validated qRT-PCR for *kissgnrh*, and *β-actin* mRNAs [17,20]. The PCR reaction mixture (20 μl) contained 1X Brilliant II Fast SYBR Green QPCR Master Mix, 0.1 μM each of forward and reverse primer, and 1 μl cDNA sample. For negative control, cDNA sample was replaced with autoclaved distilled water. Duplicate reactions were performed for the standards, target and reference genes, from 5–6 fish collected per ovarian stage. The amounts of target and endogenous reference gene in experimental samples were determined from the respective standard curves using MxPrO QPCR Software. Transcript levels of *kiss* and *gnrh* mRNAs were normalized to the levels of *β-actin*; the data are expressed as relative mRNA levels. Based on two qRT-PCR assays, the intra- and interassay coefficients of variation (CV) for *kiss* and *gnrh* mRNAs were less than 8%. All qRT-PCR assays were conducted where practically possible according to the MIQE (Minimum Information for Publication of qRT-PCR experiments) guidelines by Bustin et al. [25].

**Table 2:** **List of primers used for real-time PCR expression analysis of*kiss*and*gnrh*mRNAs**

**Primer name**	**Nucleotide sequences (5′-3′)**
Mac. Kiss1 RT Fw	CTACGACTCCTTGTTGCTTTG
Mac. Kiss1 RT Rv	TGATCTTCACTGTAGTTGGTGG
Mac. Kiss2 RT Fw	CTGAACAGAGGACACAAGGAAG
Mac. Kiss2 RT Rv	CTCAGGCTGAAACAAAGGTTAG
Mac. RT sbGnRH Fw	GCTGCTTCTTGGATCAGTAGTG
Mac. RT sbGnRH Rv	AACCCCTCAACTACATCATCC
Mac. RT cGnRH-II Fw	TGGGGTTGCTTCTATGTGTG
Mac. RT cGnRH-II Rv	TCCTCTGAAATCTCTGGTGTG
Mac. RT sGnRH Fw	ACTGGTCCTATGGATGGCTAC
Mac. RT sGnRH Rv	TTCAGGAAGAGACACCACACC

### Time-resolved fluoroimmunoassay analysis of GnRH peptides in the brain and pituitary

Brain and pituitary extracts were prepared following the protocol described earlier [26,27]. Brain and pituitary GnRH peptide levels were measured using a previously developed time-resolved fluoroimmunoassay (TR-FIA) system to quantify levels of GnRH1 (sbGnRH form), GnRH2 (cGnRH-II form), and GnRH3 (sGnRH form) in tissue extracts [28,29]. Parallelism between the typical standard curves of each GnRH peptide and the corresponding competition curves of sample extracts of chub mackerel was confirmed with serially two-fold-diluted standards and sample extracts in TR-FIA assay buffer [20]. The detection range, minimum detectable limit, and cross reactivity data are presented in our recent publication [20]. The intra- and interassay CV values of TR-FIA for GnRH1 were 9.0% and 19.6%, those for GnRH2 were 7.5% and 5.8%, and those for GnRH3 were 7.4% and 10.3%. GnRH peptide levels in the brain and pituitary samples are expressed as ng/mg tissue and ng/pituitary, respectively.

### Data analysis

All data are represented as the mean ± standard error of the mean. Changes in the levels of *kiss* and *gnrh* mRNAs in the brain and GnRH peptides in the brain and pituitary during different ovarian stages were analyzed by one-way ANOVA, followed by Tukey’s multiple comparison test. p<0.05 was considered significant and different letters in figures represent significant differences between different ovarian stages. All analyses were conducted in GraphPad Prism4.

## Results

### Ovarian histology

**Figure 1:**
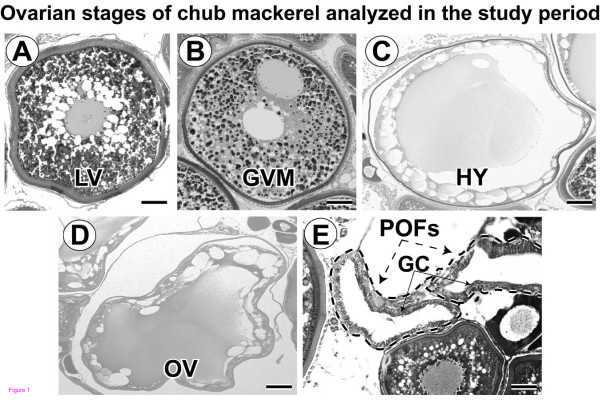
**Histological images of ovarian stages of chub mackerel analyzed in the study period.** (**A**) Late vitellogenesis (LV). (**B**) Germinal vesicle migration (GVM). (**C**) Hydration (HY). (**D**) Ovulation (OV). (**E**) Post-ovulation (POV); dashed lines indicate presence of post-ovulatory follicles (POFs) with hypertrophied granulosa cells (GC). Scale bars = 100 μm.

The histological images of different ovarian stages analyzed in the present study are presented in Figure 1. LV stage oocytes (Figure 1A) were characterized by the presence of yolk globules around centrally located germinal vesicle of first clutch late vitellogenic oocytes. In the GVM stage oocytes (Figure 1B), GV migration to the animal pole was observed with one or two continuous masses of yolk in the central region of the oocyte. HY stage oocytes (Figure 1C) were transparent and enlarged after germinal vesicle breakdown. OV stage (Figure 1D) was characterized by the presence of freshly ovulated eggs in the ovarian cavity. POV stage (Figure 1E) showed the presence of 6- to 8-h old post-ovulatory follicles (POFs) in the ovarian tissue, characterized by hypertrophied follicular granulosa cells [30].

### Changes in *kiss1* and *kiss2* mRNA levels in the brain

**Figure 2:**
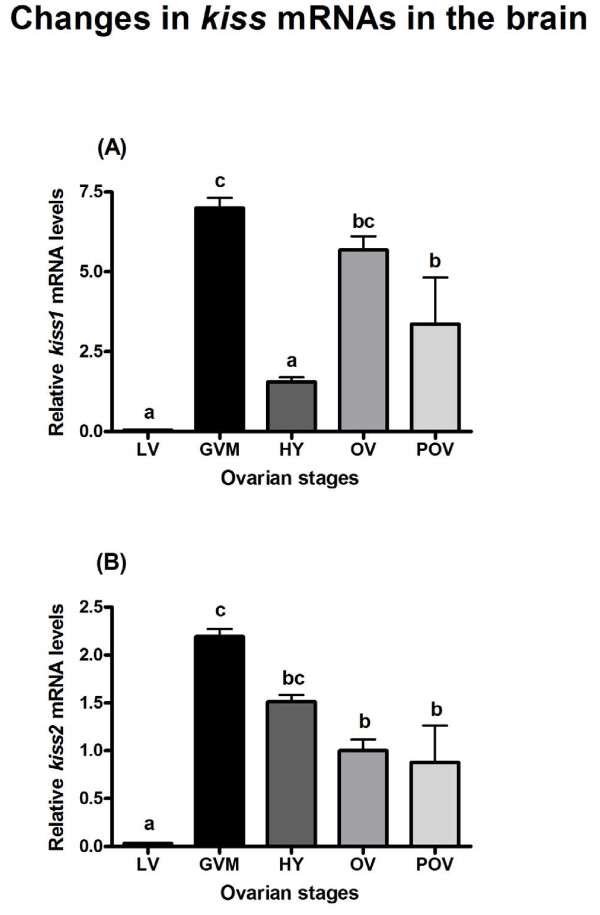
**Changes in*kiss1*(A) and*kiss2*(B) mRNA levels in the brain of adult chub mackerel during different stages of spawning cycle.** Each bar represents mean ± SEM from 5–6 fish per ovarian stage (Refer Table 1). Different letters above the bars represent significant differences (p<0.05) between stages. LV, late vitellogenesis; GVM, germinal vesicle migration; HY, hydration; OV, ovulation; POV, post-ovulation.

The levels of *kiss1* mRNA significantly increased from the LV stage to GVM (P<0.001); declined during HY and then increased during the OV and POV (P<0.001 for OV and P<0.05 for POV) periods (Figure 2A). Similar to *kiss1*, *kiss2* mRNA levels significantly increased during the GVM stage (P<0.001). However, *kiss2* mRNA levels declined during the OV and POV periods (P<0.01 for OV and P<0.001 for POV; Figure 2B).

### Changes in *gnrh1*, *gnrh2*, and *gnrh3* mRNA levels in the brain

**Figure 3:**
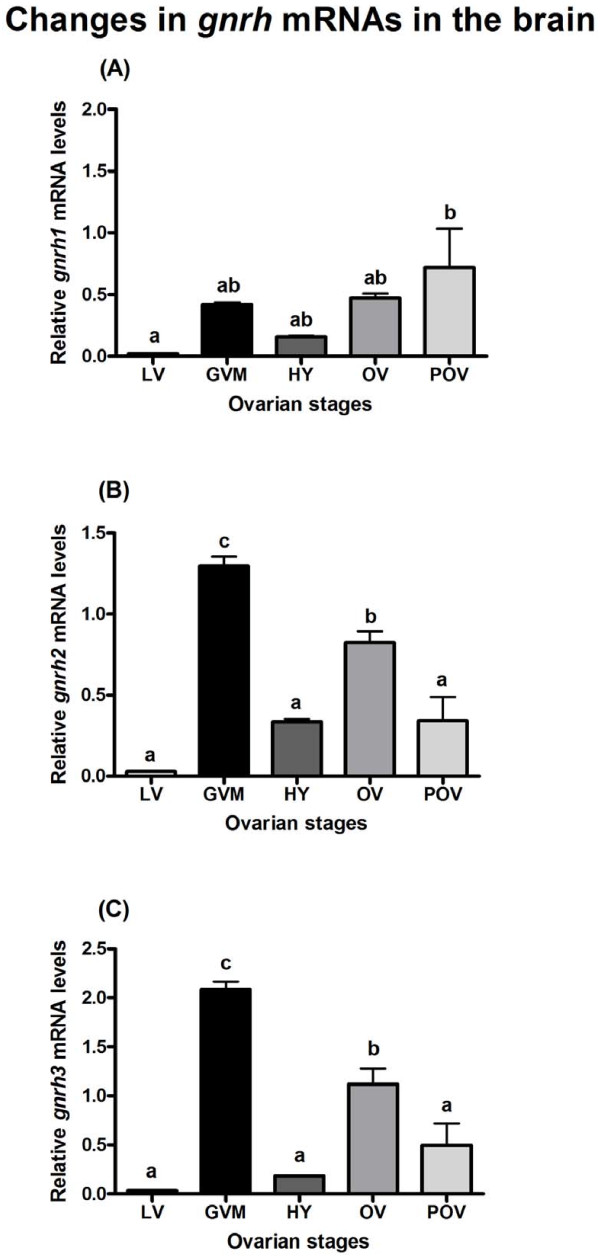
**Changes in mRNA levels of*gnrh1*(A),*gnrh2*(B), and*gnrh3*(C) in the brain of adult chub mackerel during different stages of spawning cycle.** Each bar represents mean ± SEM from 5–6 fish per ovarian stage (Refer Table 1). Different letters above the bars represent significant differences (p<0.05) between stages. LV, late vitellogenesis; GVM, germinal vesicle migration; HY, hydration; OV, ovulation; POV, post-ovulation.

The levels of *gnrh1* mRNA showed significant increase during the POV period (P<0.05; Figure 3A). However, *gnrh2* and *gnrh3* mRNA levels significantly increased during the GVM period (P<0.001) and then decreased during HY period (P<0.001). Again, their levels increased during OV period and then decreased during POV period (Figure 3B,C).

### Changes in GnRH1, GnRH2, and GnRH3 peptide levels in the brain and pituitary

**Figure 4:**
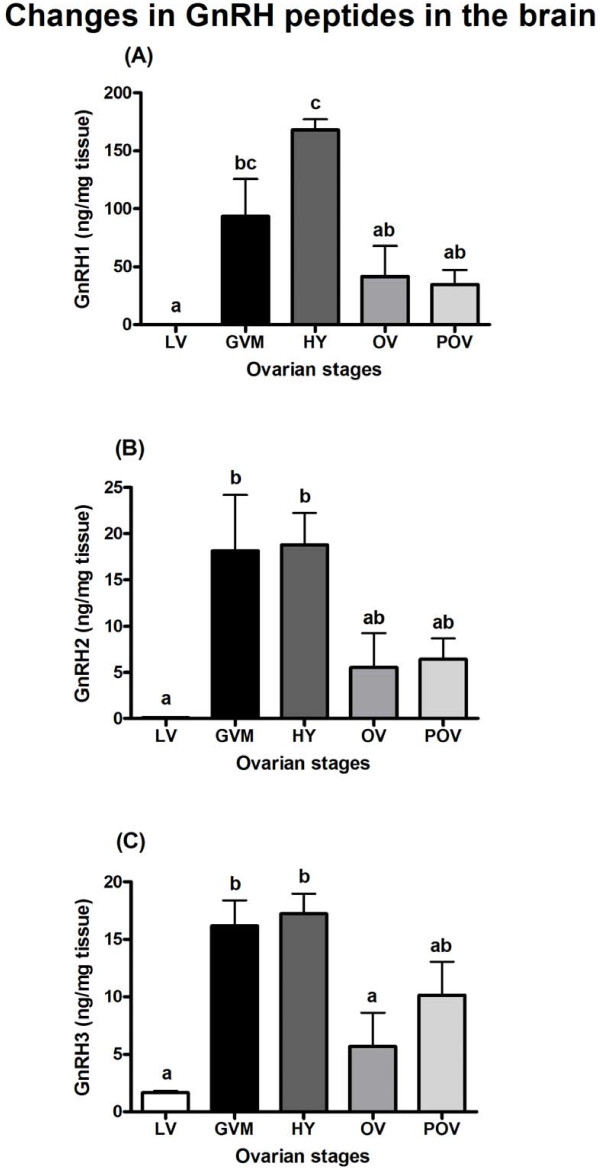
**Changes in peptide levels of GnRH1 (A), GnRH2 (B), and GnRH3 (C) in the brain of adult chub mackerel during different stages of spawning cycle.** Each bar represents mean ± SEM from 4–6 fish per ovarian stage (Refer Table 1). Different letters above the bars represent significant differences (p<0.05) between stages. LV, late vitellogenesis; GVM, germinal vesicle migration; HY, hydration; OV, ovulation; POV, post-ovulation.

GnRH1, GnRH2, and GnRH3 peptide levels in the brain were significantly elevated during the GVM (P<0.05) and HY (P<0.001) periods (Figure 4A,B,C). GnRH1 levels significantly declined during OV and POV periods (P<0.01; Figure 4A); GnRH2 levels were low during OV and POV periods but did show any significant differences with HY levels (Figure 4B); GnRH3 levels significantly declined during OV (P<0.05) and were low during POV periods (Figure 4C).

Pituitary peptide levels of all three GnRH forms did not show any significant differences among different ovarian stages (See Additional file 1: Figure S1).

## Discussion

The present study is part of a series of works targeted towards understanding the molecular basis of chub mackerel reproduction with the aim of correcting reproductive dysfunction in captivity [15,17,19,20,31]. In the present study, after GnRHa administration to fish, first spawning was observed 34–36 h post-injection and subsequent daily spawning occurred between 22.00 and 24.00 h till day 7 post-injection. Based on the previous reports demonstrating significant decline in the plasma concentration of GnRH agonist on day 5 after intramuscular injection with the GnRH agonist suspended in coconut oil in the Plaice [22,23], results of the present study likely indicate an endogenous profile of female chub mackerel undergoing FOM and ovulation in captivity.

It was interesting to find that both *kiss1* and *kiss2* mRNA levels in the brain peaked during FOM stage. However, it has been previously demonstrated that *kiss1* levels did not show any fluctuation and *kiss2* levels remained low during late vitellogenic and post-spawning periods in female chub mackerel [17]. These results suggest an ovarian stage dependent expression of *kiss1* and *kiss2* in the brain of chub mackerel. Similar observations on differential expression changes of *kiss* mRNAs in the brain at different reproductive stages of other teleosts were reported previously. Biran et al. [32] have found that in female zebrafish (*Danio rerio*), *kiss1* mRNA levels in the brain gradually increased during the first 2–8 weeks of life to peak in fish with large mature vitellogenic follicles at 12 weeks. Subsequently, Kitahashi et al. [33] found both *kiss1* and *kiss2* mRNA levels in the brain peaked 30 days after fertilization and remained high during puberty and adulthood. In grass pufferfish (*Takifugu niphobles*), expressing only *kiss2*, mRNA levels peaked in the brain and pituitary of adult mature and spawning females [34]. Similarly, in the brain of mature female striped bass (*Morone saxatilis*), both *kiss1* and *kiss2* mRNAs, including levels of their receptors *gpr54-1* and *gpr54-2*, were found to be significantly increased in comparison to juvenile and prepubertal fish [35]. In female Senegalese sole (*Solea senegalensis*), Mechaly et al. [36] found highest *kiss2* mRNA expression in the forebrain and midbrain either before or during the spawning season. However, in Atlantic cod (*Gadus morhua*), *kiss2* mRNA expression in the brain was elevated in the vitellogenic females [37]. Based on these results, we hypothesize that increased *kiss* mRNA levels in the brain are likely involved in the regulation of FOM and ovulation in chub mackerel. Future studies on the investigation of *kiss* expression in the brain of naturally spawning female chub mackerel will help to clarify the proposed hypothesis.

Recent studies suggest that the expression of kisspeptin receptor appears to have a more critical role in regulating the reproductive processes than its ligand [38]. In sheep, administration of kisspeptin decapeptides (Kiss1-10) was shown to regulate expression of kisspeptin receptors in the brain [39]. Similarly, in a prepubertal yellowtail kingfish (*Seriola lalandi*), administration of Kiss2-10 showed a significant dose-dependent response in the relative mRNA expression of kisspeptin receptor (*Kiss2r*) [38]. Interestingly, in zebrafish, habenula *kiss1* neurons were shown to coexpress kisspeptin receptor (*kissr1*) [33,40]. Further, administration of Kiss1-10 was shown to decrease habenula *kiss1* mRNA expression, suggesting autocrine regulation of the *kiss1* gene in the zebrafish [40]. In light of the above, we have recently isolated two isoforms of kisspeptin receptors from the brain of chub mackerel (Ohga et al., unpublished observations). Future analyses on the expression changes of kisspeptin receptors at different reproductive stages and ligand-receptor binding affinity will help to further clarify the role of kisspeptin system in the control of reproductive processes in chub mackerel. Recently, for the first time in fish, Beck et al. [41] revealed that exogenous administration of kisspeptin peptides has potential to accelerate gonadal development in the basses of the family Moronidae, and their hybrid. In line with the above report, functional studies of the effects of kisspeptin peptides on inducing gonadal growth and maturation in chub mackerel merits investigation.

Chub mackerel show asynchronous type of ovarian development, containing two or three clutches of vitellogenic oocytes of different diameters [42]. During spawning season, only a small percentage of first clutch late vitellogenic oocytes undergo FOM, hydration, ovulation, and spawning with successive progression of mid and early vitellogenic oocytes [16,43]. Interestingly, *kiss1* but not *kiss2* expression in the brain was found to increase during ovulatory and post-ovulatory periods, when the second clutch of vitellogenic oocytes is likely to undergo FOM on the following day (See Additional file 2: Figure S2). Our previous study [17] showed that during late vitellogenic and post-spawning periods, *kiss2* but not *kiss1* expression in the brain decreased. The post-spawning period analyzed in our previous study [17] corresponds to termination of spawning season (August) and ovaries contain mainly atretic oocytes with degenerated late-vitellogenetic oocytes. In contrast, the post-ovulatory period analyzed in the present study corresponds to spawning season (April-June) and ovaries contain post-ovulatory follicles with two or three clutches of vitellogenic oocytes. Moreover, it is likely that these fish undergo repetitive spawning activity before termination of spawning season. These results suggest differential expression changes of *kiss1* and *kiss2* in the brain in response to unknown factors. The gonadal sex steroids have been demonstrated to act in the regulation of kisspeptin expression in the brain of mammals and teleosts [44,45]. For instance, among teleost fishes expressing two *kiss* forms (*kiss1* and *kiss2*), ovarian estrogen has been shown to regulate region specific *kiss* expression in the brain. In the brain of medaka (*Oryzias latipes*), only *kiss1* neurons in the nucleus ventral tuberis (NVT) have shown to be up-regulated by ovarian estrogen [46,47]. However, in the brain of goldfish (*Carassius auratus*), only *kiss2* neurons in the preoptic area were shown to be up-regulated by ovarian estrogen [48]. Interestingly, in the prepubertal zebrafish, estradiol treatment was shown to enhance expression of both *kiss1* and *kiss2*[49]. Future studies on the localization of *kiss* expression in the brain and role of sex steroids on the regulation of *kiss* expression will help to further define the significance of differential expression changes of *kiss1* and *kiss2* in the brain of chub mackerel.

Presently, for chub mackerel there is no anatomical evidence to show that kisspeptin system is directly or indirectly involved in the regulation of GnRH neurons. For the first time, Parhar et al. [7] demonstrated coexpression of kisspeptin receptor in GnRH1, GnRH2, and GnRH3 neurons in Nile tilapia (*Oreochromis niloticus*). Subsequently, Nocillado et al. [50] found a positive correlation in the brain expression pattern of kisspeptin receptor and GnRH in female grey mullet (*Mugil cephalus*). In zebrafish, kisspeptin immunoreactive axonal fibers were shown to interact with hypophysiotropic GnRH3 neuronal systems [51]. Also, in the brain of female red seabream, expression changes of *kiss2* mRNA were shown to correlate with number of GnRH1-immunoreactive neurons [52]. Recently, in striped bass, kisspeptin receptor was colocalized in GnRH1 neurons, indicating direct influence of kisspeptin on regulation of GnRH1 neuronal system [35]. In the present study, we found that an increase in *kiss1* and *kiss2* mRNA levels coincided with an increase in *gnrh2* and *gnrh3* levels in the brain, including the peptide levels of all three GnRH forms during FOM. These findings, including the data of other studies, suggest that the role of kisspeptins in the regulation of GnRH neuronal system is likely conserved in the chub mackerel and merits future investigation on the colocalization of kisspeptin and GnRH system.

In several teleosts, either mRNA or peptide levels of the hypophysiotropic GnRH form, i.e., GnRH1 in teleosts expressing three GnRH forms, and mainly GnRH3 in the case of those expressing two forms, have been shown to fluctuate significantly during ovarian maturation or spawning season [10,53]. However, in the brain of gilthead seabream expressing three GnRH forms and showing asynchronous type of ovarian development as that of chub mackerel, elevation in the levels of all three GnRH mRNAs and plasma LH were found 8h before spawning, when the germinal vesicle was located next to the micropyle of the oocyte [21]. This is in agreement with the findings of present study showing elevations in the levels of *gnrh2* and *gnrh3* mRNAs, including the peptide levels of GnRH1, GnRH2, GnRH3 forms in the brain during FOM stage, germinal vesicle migration (7-10h before spawning). In the chub mackerel, we did not find any significant rise in *gnrh1* mRNA levels except during post-ovulatory period. However, it is interesting to note that this increase also coincided with an increase in the mRNA levels of pituitary gonadotropin subunits (*gpαfshβlhβ*) analyzed in the same fish samples [15]. These results suggest that the stimulatory signal of *gnrh1* contributing to increased pituitary *lhβ* levels resulting in FOM, would have initiated 14-17h before spawning in the chub mackerel. Since GnRH1 peptide levels were many-fold higher in the brain and pituitary of female chub mackerel during the FOM and ovulation stages, we propose GnRH1 form as the predominant regulator of maturation and spawning in chub mackerel. This is further supported by our previous data showing a dominant role of GnRH1 form in the regulation of vitellogenesis in chub mackerel [20].

## Conclusions

The present study revealed significant fluctuations in the levels of two *kiss* mRNAs in the brain during the FOM and ovulatory periods. Further, levels of all three *gnrh* mRNAs and their peptides in the brain were found to fluctuate during FOM and ovulatory periods. These results indicate increased expression of multiple Kiss and GnRH forms in the brain and suggest their possible involvement in the regulation of FOM and ovulation in captive female chub mackerel. Future studies on the expression of *kiss* and *gnrh* mRNAs and changes in their peptide levels in the brain of naturally spawning female chub mackerel will be important to understanding their role in the reproductive dysfunction of captive fish.

## Competing interests

The authors declare that they have no competing interests.

## Authors’ contributions

SS and MM was responsible for the experimental design. SS was the principal writer of the manuscript and contributed to the mRNA expression analysis, data analysis, and interpretation of the results. HK, MY, and HO assisted in fish and tissue sampling. MA was responsible for GnRH peptide analyses. AY, AS, and MM contributed to the data interpretation and supervised this work. All authors read and approved the final manuscript.

## Supplementary Material

Additional file 1 **Figure S1.** Changes in peptide levels of GnRH1 (A), GnRH2 (B), and GnRH3 (C) in the pituitary of adult chub mackerel during different stages of spawning cycle. Each bar represents mean ± SEM from 4–6 fish per ovarian stage (Refer Table 1). Different letters above the bars represent significant differences (p<0.05) between stages. LV, late vitellogenesis; GVM, germinal vesicle migration; HY, hydration; OV, ovulation; POV, post-ovulation. (JPEG 673 kb) Click here for file

Additional file 2 **Figure S2.** Summarized figure showing expression changes of *kiss1* (red line), *kiss2* (blue line), *gnrh1* (pink line), *gnrh2* (green line), *gnrh3* (purple line) mRNAs in the brain; GnRH1 (pink break line), GnRH2 (green break line), GnRH3 (purple break line) peptides in the brain and pituitary; *gpα* (brown line), *fshβ* (yellow orange line), *lhβ* (orange line) mRNAs (reported previously by Nyuji et al. [15]) in the pituitary of chub mackerel (*Scomber japonicus*) at different ovarian stages analyzed in the present study. (JPEG 863 kb) Click here for file
