# Supplementary figures and images for: Increased expression of kisspeptin and GnRH forms in the brain of scombroid fish during final ovarian maturation and ovulation
Source: Reprod Biol Endocrinol. 2012 Aug 27;10:64. doi: 10.1186/1477-7827-10-64 (PMC3453514; doi:10.1186/1477-7827-10-64)

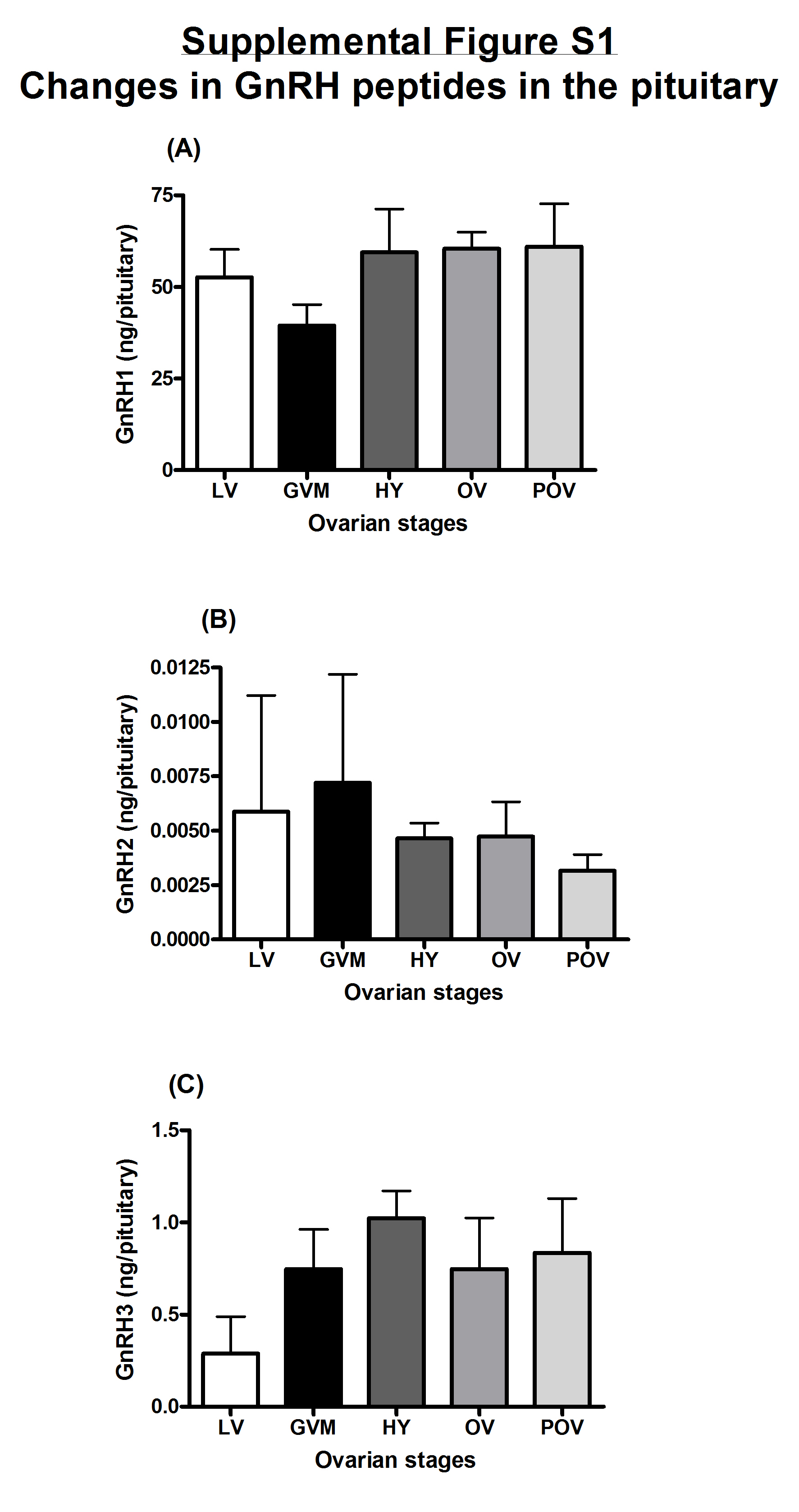

Supplement: Additional file 1 — Figure S1. Changes in peptide levels of GnRH1 (A), GnRH2 (B), and GnRH3 (C) in the pituitary of adult chub mackerel during different stages of spawning cycle. Each bar represents mean ± SEM from 4–6 fish per ovarian stage (Refer Table 1). Different letters above the bars represent significant differences (p<0.05) between stages. LV, late vitellogenesis; GVM, germinal vesicle migration; HY, hydration; OV, ovulation; POV, post-ovulation. (JPEG 673 kb) [file 1477-7827-10-64-S1.jpeg]

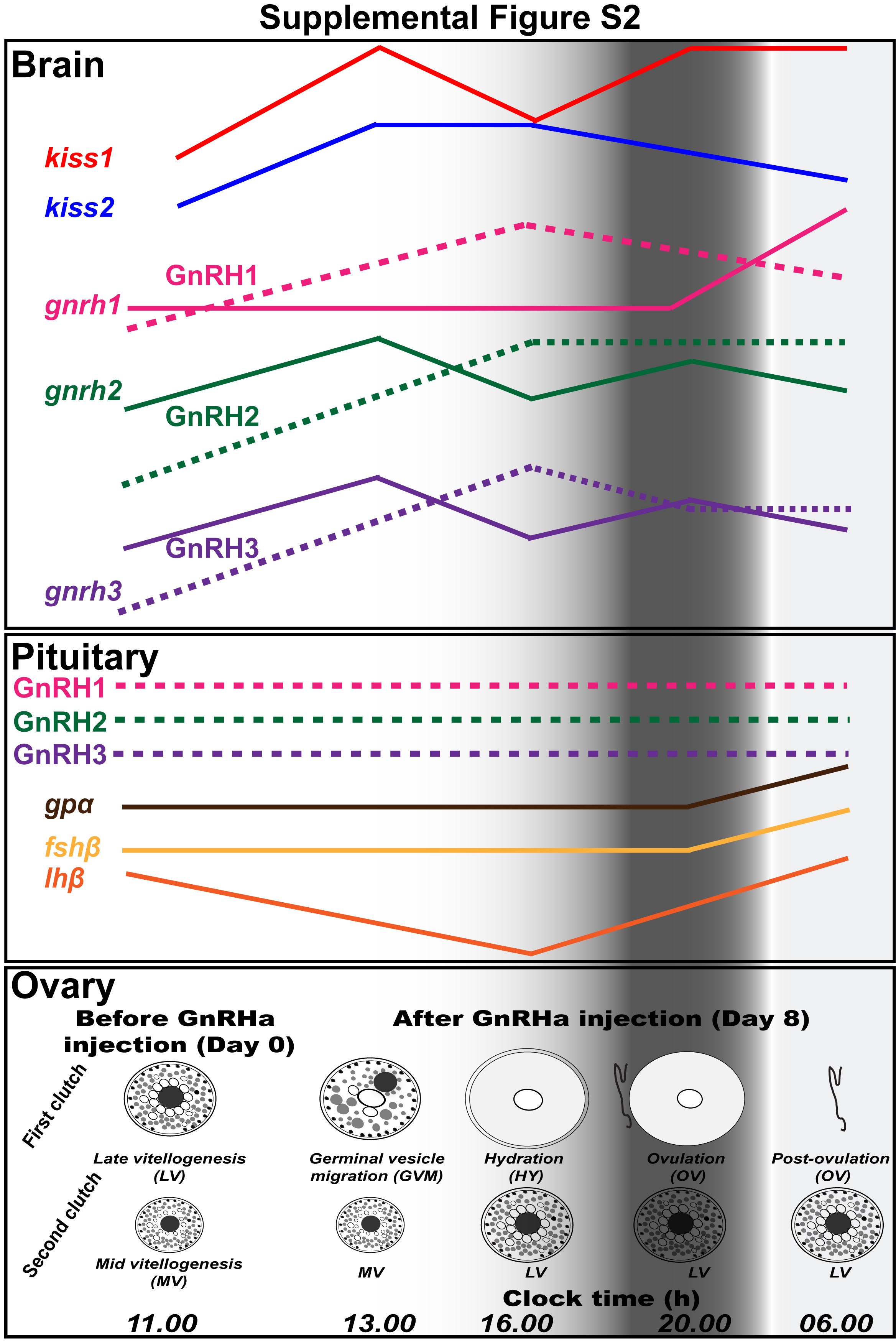

Supplement: Additional file 2 — Figure S2. Summarized figure showing expression changes of kiss1 (red line), kiss2 (blue line), gnrh1 (pink line), gnrh2 (green line), gnrh3 (purple line) mRNAs in the brain; GnRH1 (pink break line), GnRH2 (green break line), GnRH3 (purple break line) peptides in the brain and pituitary; gpα (brown line), fshβ (yellow orange line), lhβ (orange line) mRNAs (reported previously by Nyuji et al. [15]) in the pituitary of chub mackerel (Scomber japonicus) at different ovarian stages analyzed in the present study. (JPEG 863 kb) [file 1477-7827-10-64-S2.jpeg]
